# Supplementary material for: Resolving the full spectrum of human genome variation using Linked-Reads
Source: Genome Res. 2019 Apr;29(4):635–45. doi: 10.1101/gr.234443.118 (PMC6442396; doi:10.1101/gr.234443.118)
Supplement: Supplemental Material [file supp_gr.234443.118_Supplemental_longranger-2.2.zip › longranger-2.2.tar.bz2!/lr2.2-public/lib/stan/cmdstan/stan/doc/api/html/welford__covar__estimator_8hpp_source.html]

Stan: src/stan/prob/welford\_covar\_estimator.hpp Source File


|  |  |
| --- | --- |
|  | Stan  2.5.0  probability, sampling & optimization |


- Main Page
- Related Pages
- Namespaces
- Classes
- Files

- File List
- File Members

All Classes Namespaces Files Functions Variables Typedefs Enumerations Enumerator Friends Macros Pages

- src
- stan
- prob

welford\_covar\_estimator.hpp

Go to the documentation of this file.

1 #ifndef STAN\_\_PROB\_\_WELFORD\_\_COVAR\_\_ESTIMATOR\_\_BETA

2 #define STAN\_\_PROB\_\_WELFORD\_\_COVAR\_\_ESTIMATOR\_\_BETA

3

4 #include <vector>

5 #include <stan/math/matrix/Eigen.hpp>

6

7 namespace stan {

8

9  namespace prob {

10

11  class welford\_covar\_estimator {

12

13  public:

14

15  welford\_covar\_estimator(int n): \_m(Eigen::VectorXd::Zero(n)),

16  \_m2(Eigen::MatrixXd::Zero(n, n))

17  { restart(); }

18

19  void restart() {

20  \_num\_samples = 0;

21  \_m.setZero();

22  \_m2.setZero();

23  }

24

25  void add\_sample(const Eigen::VectorXd& q) {

26

27  ++\_num\_samples;

28

29  Eigen::VectorXd delta(q - \_m);

30  \_m += delta / \_num\_samples;

31  \_m2 += (q - \_m) \* delta.transpose();

32

33  }

34

35  int num\_samples() { return \_num\_samples; }

36

37  void sample\_mean(Eigen::VectorXd& mean) { mean = \_m; }

38

39  void sample\_covariance(Eigen::MatrixXd& covar) {

40  if(\_num\_samples > 1)

41  covar = \_m2 / (\_num\_samples - 1.0);

42  }

43

44  protected:

45

46  double \_num\_samples;

47

48  Eigen::VectorXd \_m;

49  Eigen::MatrixXd \_m2;

50

51  };

52

53  } // prob

54

55 } // stan

56

57

58 #endif

stan::prob::welford\_covar\_estimator::num\_samples

int num\_samples()

**Definition:** welford\_covar\_estimator.hpp:35

stan::prob::welford\_covar\_estimator::\_m

Eigen::VectorXd \_m

**Definition:** welford\_covar\_estimator.hpp:48

stan::prob::welford\_covar\_estimator::sample\_covariance

void sample\_covariance(Eigen::MatrixXd &covar)

**Definition:** welford\_covar\_estimator.hpp:39

stan::prob::welford\_covar\_estimator::\_num\_samples

double \_num\_samples

**Definition:** welford\_covar\_estimator.hpp:46

stan::math::mean

boost::math::tools::promote\_args< T >::type mean(const std::vector< T > &v)

Returns the sample mean (i.e., average) of the coefficients in the specified standard vector...

**Definition:** mean.hpp:23

Eigen.hpp

stan::prob::welford\_covar\_estimator::\_m2

Eigen::MatrixXd \_m2

**Definition:** welford\_covar\_estimator.hpp:49

stan::prob::welford\_covar\_estimator::welford\_covar\_estimator

welford\_covar\_estimator(int n)

**Definition:** welford\_covar\_estimator.hpp:15

stan::prob::welford\_covar\_estimator

**Definition:** welford\_covar\_estimator.hpp:11

stan::prob::welford\_covar\_estimator::sample\_mean

void sample\_mean(Eigen::VectorXd &mean)

**Definition:** welford\_covar\_estimator.hpp:37

stan::prob::welford\_covar\_estimator::restart

void restart()

**Definition:** welford\_covar\_estimator.hpp:19

stan::prob::welford\_covar\_estimator::add\_sample

void add\_sample(const Eigen::VectorXd &q)

**Definition:** welford\_covar\_estimator.hpp:25


---

[ Stan Home Page ]

*© 2011–2014,
Stan Development Team.*
